# Experiences of reduction and discontinuation of antipsychotics: a qualitative investigation within the RADAR trial

**DOI:** 10.1016/j.eclinm.2023.102135

**Published:** 2023-09-28

**Authors:** Nicola Morant, Maria Long, Sandra Jayacodi, Ruth Cooper, Johura Akther-Robertson, Jacki Stansfeld, Mark Horowitz, Stefan Priebe, Joanna Moncrieff

**Affiliations:** a Division of Psychiatry, University College London, London, UK; b Health Services Research & Management, City University, London, UK; c Research & Development Department, North East London NHS Foundation Trust, London, UK; d Service User and Independent Peer Researcher, UK; e Unit for Social and Community Psychiatry, Queen Mary University of London, London, UK; f East London NHS Foundation Trust, Newham Centre for Mental Health, London, UK; g NIHR Mental Health Policy Research Unit, Institute of Psychiatry, Psychology & Neuroscience, King’s College London, London, UK

**Keywords:** Mental health, Antipsychotics, Qualitative, Psychosis, Experiences, Medication optimisation

## Abstract

**Background:**

Antipsychotics are a core treatment for psychosis, but the evidence for gradual dose reductions guided by clinicians is under-developed. The RADAR randomised controlled trial (RCT) compared antipsychotic reduction and possible discontinuation with maintenance treatment for people with recurrent psychotic disorders. The current study explored participants’ experiences of antipsychotic reduction or discontinuation within this trial.

**Methods:**

This qualitative study was embedded within the RADAR RCT (April 2017–March 2022) that recruited 253 participants from specialist community mental health services in 19 public healthcare localities in England. Participants were adults with recurrent non affective psychosis who were taking antipsychotic medication. Semi-structured interviews, lasting 30–90 min, were conducted after the trial final 24-month follow-up with 26 people who reduced and/or discontinued antipsychotics within the trial, sampled purposively for diversity in sociodemographic characteristics, trial variables, and pre-trial medication and clinical factors. Data were analysed using thematic analysis and findings are reported qualitatively.

**Findings:**

Most participants reported reduced adverse effects of antipsychotics with dose reductions, primarily in mental clouding, emotional blunting and sedation, and some positive impacts on social functioning and sense of self. Over half experienced deteriorations in mental health, including psychotic symptoms and intolerable levels of emotional intensity. Nine had a psychotic relapse. The trial context in which medication reduction was explicitly part of clinical care provided various learning opportunities. Some participants were highly engaged with reduction processes, and despite difficulties including relapses, developed novel perspectives on medication, dose optimisation, and how to manage their mental health. Others were more ambivalent about reduction or experienced less overall impact.

**Interpretation:**

Experiences of antipsychotic reductions over two years were dynamic and diverse, shaped by variations in dose reduction profiles, reduction effects, personal motivation and engagement levels, and relationships with prescribers. There are relapse risks and challenges, but some people experience medication reduction done with clinical guidance as empowering. Clinicians can use findings to inform and work flexibly with service users to establish optimal antipsychotic doses.

**Funding:**

10.13039/501100000272National Institute for Health Research.

**Research in context**

**Evidence before this study**

Existing qualitative research suggests that while taking antipsychotic medication is experienced as beneficial for symptom management and relapse prevention, many people describe a high burden of adverse effects or are unhappy about the prospect of taking antipsychotics long-term. However, there is little qualitative research about experiences of reducing or discontinuing antipsychotics. In January 2023, we updated a previous systematic search for studies about experiences of antipsychotic medication using terms to capture experiences, attitudes, opinions or personal accounts of antipsychotic medication, and adding ("reduction" OR "discontinuation" OR "withdrawal" or "stop∗") AND ("qualitative" OR "thematic"). This identified 56 relevant studies, including a recent systematic review of experiences of psychiatric medication discontinuation, within which four studies involved antipsychotics. Overall, most studies focused primarily on how and why people make decisions about antipsychotic discontinuation, and all involved people who had actively chosen to reduce or discontinue medication usually in a pre-planned way, with or without the knowledge or support of clinicians. Reducing or stopping medication was often linked with social functioning or psychological benefits but described as a challenging process requiring determination and social support. No studies investigated antipsychotic reduction or discontinuation within a randomised controlled trial (RCT) or any other structured programme.

**Added value of this study**

To our knowledge, this is the first qualitative study to explore experiences of reducing or discontinuing antipsychotic medication when this was overseen by clinicians within an RCT designed to assess reduction/discontinuation outcomes. Our study accesses a broader sample than previous studies, including people who were open to medication reduction but may not otherwise have chosen or initiated this themselves. The trial context also enabled exploration of gradual reduction experiences over a consistent 24-month period. Findings mirror those of previous studies on reduction/discontinuation done principally independent of clinicians in showing a variable profile of multiple experiences covering reduced adverse effects, improved social functioning, emotional difficulties, mental health deteriorations and relapses. These experiences provided a valuable learning opportunity about medication and mental health management for many participants, some of whom showed high levels of engagement and ownership of reduction process, although others were less engaged. Because reduction was clinically sanctioned, we were able to explore how these processes related to relationships with clinicians.

**Implications of all the available evidence**

Antipsychotic use, management and optimisation is a complex and controversial topic about which clinicians and service users need clearer knowledge, evidence and guidance. The emergent evidence-base about experiences and first-person reported impacts of reducing or discontinuing antipsychotics can usefully complement trial-based evidence and inform clinical guidelines. Evidence shows that experiences of reducing antipsychotic medication are highly variable and often challenging. Service users and clinicians considering reduction should anticipate risks of mental health deteriorations alongside potential benefits. The process should be person-led and flexible with reductions made slowly and with careful oversight. Open partnerships with clinicians and psychosocial support are recommended.

## Introduction

Long-term use of antipsychotic medication is recommended to prevent relapse in those with recurrent psychosis.^1^ Qualitative research suggests that whilst people value antipsychotics for symptom management and relapse prevention, they often experience adverse effects that can impair social functioning.^2^^,^^3^ Many are ambivalent about long-term use and would like to try dose reduction or medication discontinuation.^4^ However, support for this is often limited as clinicians may feel ill-equipped to guide reduction processes, lack evidence-based guidelines or have concerns about risk.^5^^,^^6^

Although it is known that many people reduce or discontinue antipsychotics without the support or knowledge of prescribers,^7^ much less is known about the processes, personal and mental health impacts, and experiences involved. A small number of qualitative studies of people who have reduced or stopped antipsychotics, mostly independent of clinicians, highlight how this is often part of a process of self-determination.^8^, ^9^, ^10^, ^11^ Context, levels of social support and coping strategies to support well-being and manage physiological and psychological challenges have been identified as important determinants of the experience and success of reduction processes.^8^, ^9^, ^10^, ^11^, ^12^ However, as reduction strategies may vary from abrupt discontinuation to dose reductions over months or years, it is difficult to draw conclusions from this small body of work. No studies have investigated how gradual reduction/discontinuation of antipsychotics is experienced when clinically guided and offered to people who might not otherwise have initiated it. The RADAR randomised controlled trial (RCT) compared antipsychotic reduction and possible discontinuation with maintenance treatment in people with schizophrenia or other non-affective psychotic disorders.^13^ Here we report an embedded qualitative study. The broad research question guiding this work was to explore participants’ subjective experiences of antipsychotic reduction/discontinuation in the trial.

## Methods

### Reporting and ethics

Methods are reported according to COREQ guidelines.^14^ A 32-item checklist showing further methodological details is provided in Appendix 1. Ethics approval was obtained (London-Brent Ethics committee, 16/LO/1507). All participants provided informed consent.

### Study design and participants

Participants were sampled purposively from the RADAR trial intervention group to obtain a diverse sample. This took account of self-reported socio-demographic characteristics (age, ethnicity, gender, employment status); trial variables (reduction profile, relapse, trial site, treating clinician); and pre-trial medication and clinical factors (time in contact with mental health services, oral/injections, clozapine use). Given the diversity of experiences accessed by this sampling strategy, we anticipated collecting data from 20 to 30 trial participants. The decision to end data collection was based on reviewing interviewee characteristics in relation to our sampling criteria, and the overall information power of the data corpus.^15^

The RADAR RCT recruited 126 people to receive a reduction intervention in which treating psychiatrists oversaw gradual and flexible reduction of antipsychotics and possible discontinuation, based on a reduction protocol over a 24-month period. Recruitment was from mental health services across England. Participants were adults with recurrent non affective psychosis, taking antipsychotic medication (excluding those who had suffered a mental health crisis or been hospitalised in the last month; who posed a serious risk as judged by treating clinicians; or were legally mandated to take medication). Details of the study protocol and findings are provided elsewhere.^13^^,^^16^

### Procedures

A semi-structured interview guide was developed by the research team with input from people with lived experience of psychosis and antipsychotics. Open-ended questions explored experiences of dose reductions/discontinuation, impacts on daily life, changes in mental health, sources of support, and views on future antipsychotic use. See Appendix 2 for the full interview schedule. Participants were contacted after final trial data collection. Before the interview, participants received broad topic prompts and a timeline of their antipsychotic use during the trial to aid recall. Interviews were conducted by study researchers and a lived experience researcher (ML, JAR, SJ, RC) in person, using video software or by telephone. Reflexive summary notes were made after each interview and shared among the team to inform data collection and analytic processes. Interviews were audio-recorded, transcribed and anonymised.

### Data analysis

Data were analysed using codebook thematic analysis^17^ within NVivo software. Codebook thematic analysis was chosen because it enables a structured approach to the iterative development of meaning-based and descriptive themes; is suitable for large volumes of complex data and applied research questions; and is compatible with both collaborative, team-based working and a critical realist qualitative approach.^18^ We took a primarily inductive approach, although analysis was also informed by awareness of key issues in the literature and field of medication reduction. Initial codes were developed inductively from detailed reading of a sample of transcripts. Codes were gradually organised into thematic domains through an iterative process of coding further data and refining the coherence and specificity of themes. Our analytic approach generated both topic-based and meaning-based themes. Exploratory processes in late-stage analysis included investigating variations within themes and rereading the data corpus and interviewer reflexive summary notes to understand analytic concepts within complete interview narratives. Analysis was led by researcher interviewer ML with close involvement of NM and input from other authors. Four transcripts were independently coded by a lived experience researcher (SJ) who participated in analytic discussions. Throughout data collection and analysis, the research team, including all interviewers, exchanged ideas about emerging patterns and conceptual understandings. Discussions were characterised by high levels of reflexivity, with critical acknowledgement of the positioning of researchers within the RADAR study team.

### Role of the funding source

The funder had no role in the study design, data collection, data Formal analysis, data interpretation, or report writing.

## Results

**Fig. 1:**
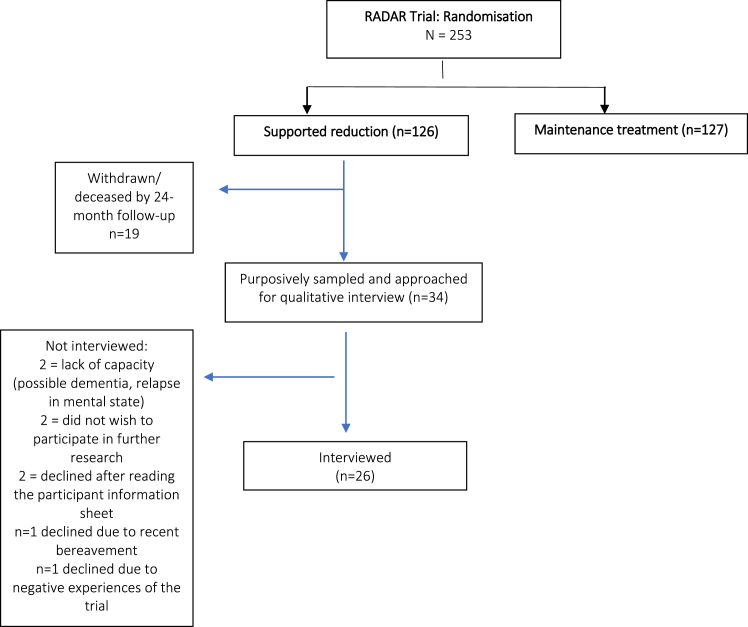
Flowchart of study participant recruitment.

**Fig. 2:**
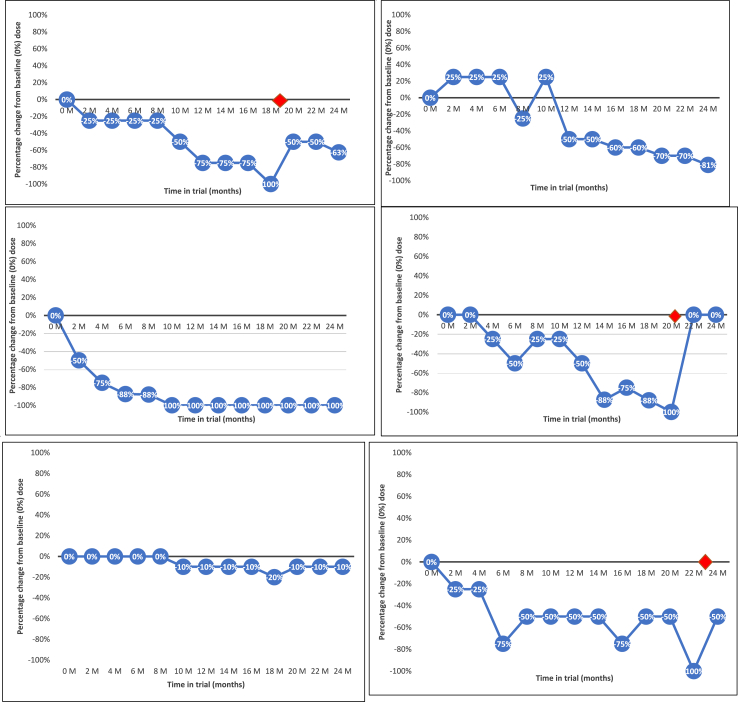
Example antipsychotic reduction profiles. Red diamond indicates relapse.

Between December 2019 and December 2021, 34 people were approached for participation in the current study, of whom 26 were interviewed (see Fig. 1). Interviews lasted 30–90 min. Table 1 shows participants’ sociodemographic and clinical characteristics, and antipsychotic reductions at trial end. Participants were drawn from 11 of the 19 public healthcare organisations where the RADAR trial was conducted. Reduction profiles during the trial varied—dose reductions were often followed by increases or periods of stabilisation as negotiated with prescribers (and permitted within the trial protocol; see Fig. 2 for illustrative examples). These diverse profiles form the context of interview narratives.

**Table 1:** Participant demographic and clinical characteristics, antipsychotic reductions and relapses (N = 26).

Characteristic	N
Primary diagnosis	
Schizophrenia	18
Other psychosis	8
Age	
<30	3
30–39	3
40–49	11
50–59	6
>60	3
Ethnicity	
White	20
Black	6
Gender	
Female	11
Male	15
Employment status	
Unemployed	18
Employed	8
Clozapine	
Yes	3
No	23
Antipsychotic polypharmacy	
Yes	6
No	20
Antipsychotic administration	
Oral	19
Depot	7
Mental health service contact (years)	
<4	3
4–10	8
11–15	3
16–20	4
20+	8
Clinician delivering RADAR reduction intervention	
RADAR affiliated clinician	9
Locality psychiatrist	14
Locality psychiatrist and RADAR clinician	3
Change in antipsychotic dose at trial end compared to baseline (CPZ equivalents)^a^	
No change or increase	5
Slight reduction	2
Reduction	6
Significant reduction	9
Discontinuation	4
Relapse during trial^b^	
No relapse	17
Non-severe relapse	4
Severe relapse	5

a CPZ = chlorpromazine equivalent; slight reduction = 1–20% CPZ; reduction = 21–50% CPZ; significant reduction = 51–99% CPZ.

b Relapse as defined by RADAR trial expert endpoint committee: Severe relapse = requiring acute psychiatric hospitalisation; non-severe relapse = managed by community services.

Findings are organised as follows: Section 1 describes the range of specific reported effects of antipsychotic reduction or discontinuation. Participants described a range of impacts experienced at different points in the 24-month trial period, or sometimes simultaneously. These included reductions of adverse medication effects with dose reductions, often linked in interview narratives to improvements in social functioning or impacts on sense of self; increases in the intensity of emotions that was sometimes linked to or preceded deteriorations in mental health or relapse; and short-term physical or psychological difficulties following dose reductions. Section 2 explores how people made sense of and related to these reduction experiences and processes. An underlying theme is forms of learning linked to participation in the RADAR reduction intervention. These were described in relation to medication, self-management strategies, and relationships with clinicians (explored in each sub-section). There were large divergences within the sample, ranging across those who reported little overall impact of the trial, people who found reductions unhelpful or challenging, and those who felt the trial had provided a novel opportunity for learning and whose perspectives in each of the above areas shifted substantially. Table 2 provides additional illustrative quotes.

**Table 2:** Additional illustrative quotes.

**1. Effects of antipsychotic reduction/discontinuation**	
1.1 Reduction of negative effects	*“I was almost 17 stone and now they can see that I’ve come down, I’ve come down to almost 14 stone… medication it makes you put on weight and they have a problem on your heart because I was having heart problems. But now I lose all the weight now so it’s not severe like before.”* P01013 *“Difference was completely different, and without medication I can wake up any time if I’ve got a job. With medication you feel very heavy early in the morning, you cannot get up when you to work. You feel like the way you act is not that quick, and then workplace maybe supervisors, managers not happy, even normal workers not happy with you.”* P02005 *“That’s another thing that’s gone away. Not only the light-headedness, but confusion. I used to get confused a lot and [partner] said I’m talking to you and he’d say you are miles away, I get lightheaded, confused. I don’t get that anymore, I’m stronger.”* P02026 *“It [reduction of meds] was very freeing. I felt I could concentrate better and had a longer concentration span. It has a good effect on my work. I think the customers probably felt that I was more in control and it felt all more in control really… I was quite surprised because I actually thought I may feel more anxious because of medication reduction but it was actually opposite - I felt less anxious than before because I could start controlling if I was under stress, I could start to influence in my mind, in my emotions to control that better… I think it was good for my marriage as well because I initiated conversations more often.”* P07006
1.2 Returning aspects of self and social functioning	*“I’ve become normal because things I was doing, I’ve started doing again…going everywhere, phoning my friends, they’re phoning me, and we talk, and we have a laugh.”* P02035 *“I was getting myself active again and things were getting done during the day. The house was getting cleaned, I was going out shopping I’m meeting friends more… I can concentrate watching a DVD or TV programme…my walking has been better, I don’t use a stick anymore…[I’ve] lost a stone in the last year… I couldn’t believe it…that’s just spurred me on to do more things.”* P02038 *“I started functioning off the medication, I was more sociable ... I functioned as an average human being, as a normal human being. But I also had this timebomb ticking away underneath me where I feared a psychosis rebound. And a psychosis rebound did happen.”* P8003
1.3 Challenges with emotional intensity	*“The lower it has gone down the more difficult, yes, since about 3.75 [mg]… There’s a lot of regret about the past 10 to 15 years, there’s a lot of emotions about that,… about the medications, about a lot of things and I haven’t been doing anything with my life particularly and now I’m [mid 40s] and it’s kind of like starting again. It’s almost waking up from a coma…day to day I’m just trying to manage my…emotions that is my problem.”* P06015 *“I don’t know just things got on top of me again. I lost control of my feelings sort of thing for a bit I suppose.”* P1037 *“It’s quite hard being on an emotional rollercoaster, flooding the memories and that. I couldn’t stop crying”* P1014 *“It was quite nice [after discontinuation] because I started sleeping more but I still felt like lightness. I still felt really anxious sometimes like flashing and crazy out of nowhere. I didn’t really feel right…[I] just look down and don’t look at the people’s faces and eyes… I’d go to a shop and not being able to, just kind of trying to rush out of there very quickly, and then I would think that I was quite strange and that has another like… emotions coming in.”* P14002
1.4 Mental health deteriorations and relapse	*“I just got paranoid [after the first reduction]… Nearly straight away… [it affected] my family because they were worried and they was ringing me all the time. And my girlfriend she wasn’t happy… I thought people were going to kill me and they were going to break into my flat. When I reduced it, it was worse.”* P01059 *“From 15mg I was fine, 10mg I was fine… when they took me to 2.5 that was it, I was [coughs] I was losing my rage and so I got into a few street fights and things like that and this was why I was put back into hospital”* P02021 *“I started feeling anxious and a bit paranoid and that’s when they started upping it again”* P7007 *“The Radar study didn’t help me, I wanted to come off medication and reduce it but I couldn’t because I got ill […] I want to reduce it and come off the medication but I’m frightened to because if I reduce it and come off medication I get ill and then I don’t know what will happen.”* P01059
1.5 Short-term difficulties/withdrawal effects	*“I didn’t realise how it would make me feel…and what a big change it would be to what I feel now [after having stabilised on a reduced dose]. …. When I went down to 7.25 that’s when I started to get really quite bad withdrawal symptoms. It was the insomnia which was really bad. I did start to get really racing thoughts again then and severe itching all over my body, uncontrollable itching… I think at that stage it was quite difficult but I just carried on and it did settle down.”* P06015 *“From 20, 15 it wasn’t, 10 I was alright. It was just when they went from 5 to 2.5 it was very quick. It was almost like we’re just doing a quick rush job now so it was a quick thing. Now say for instance I was on 5mgs for three months and then they reduced it maybe it would have been different. Maybe my body would have had time to adjust.”* P02021
**2. Making sense of reduction experiences: the RADAR trial as a novel potential learning context**	
	*“For me I think it has been a good step to be on the Radar study because I was on 20mgs of Aripiprazole before and it was a very high dose in my opinion and my psychiatrist would never have let me get off it at all, maybe not even reduce it. This [Radar] we could we find out that a minimum dose of 7.5mgs is possible for me so it’s very positive actually and even without Radar study I always ask all the psychiatrists can I get off please. I once even did it on my own and the psychiatrist refused to still look after me because he knew I would get a relapse and he couldn’t agree to this and he right because I got a relapse.”* P07006
2.1 Medication: Learning about the implications of reduction	*"I think in the future it won't be as bad because I've already identified the triggers and I know my own self what the problem is and because it's [the next reduction] not for another two years there will be periods of stress in between where I've had to deal with stressful situations on the medication and then once you come off it you are more able to deal with stressful situations without the medication.. So I'm not looking at it as I'm coming off quickly... I do hope to come off the medication and I also do hope not to relapse again."* P02001 *“Whereas before I was a bit more rigid, like I want to be on it or I don’t want to be on it, and I think that because of the trial, reducing it slowly over the years has made me realise that I can get side effects, I can maybe get ill again, is it the drugs is it me, I’m not sure… I realise things are going to happen in my life, good things and bad things, you can’t control life, you never know what’s going to happen and periods of stress and anxiety and thinking about things like that and you think, do you know, maybe I might need something to help.”* P06015 *I: Can you think of any changes that you felt when you reduced it?* *P01040: Probably more sounds and more voices and stuff like that.* *I: What was that like?* *P01040: Just loud voices and just hearing voices and stuff like that, it was probably one or two more voices there. But the voices were being louder.* *I: Was there any kind of longer-term impact of what happened [relapse] when you came off [antipsychotics]?* *P06004: It became rooted in the past and then left behind. But obviously a warning to me to not come off medication [ ….] I just wanted to be myself and free and natural, like a free spirit sort of thing, and not to rely on medication. That’s the only reason and that’s always been my motivation for not wanting to do it. But then now I just think, well I can’t live without it.”*
2.2 Self-management	*“Reducing it each time made me feel closer to feeling that I’m capable to not have to take it. So not that it was, I don’t know, like a meter of my psychosis or something, but I feel the less medication I take in general the more I am able to find life tools to help me. The more wiser and skilful I become.”* P14002 *"I think the thing is the anxiety… and the fear of the unknown that you don't know what is going to happen if you start reducing… I had the support of my friends and my sister if anything did go wrong. I think that was partly the encouragement I had to proceed with the reduction process."* P02038 *"My family have supported me… with conversation and assistance. Conversation, moral support… I think it made it easier"* P03004 *"If I hear someone laughing I think they're laughing at me …. I try to ignore it now and just quickly walk past… obviously they are laughing at each other or something… I realise that now."* P01014 *"With the drinking I have had a couple of serious relapses and… it's just to do with the emotions, coping with emotions and with people and I'm not used to it. I find I've got quite a lot of social anxiety."* P06015
2.3 Relationships with clinicians: partnership possibilities	*“I think there was secondary voice which is Dr [name] who is taking on and trying to analyse what I was going through. So it wasn’t just my opinion now it was two people’s opinion. So rather than sitting there thinking in my head oh this is what I’ve done to try and sort out the situation or this is what I have to think to sort out the situation I could think in two ways now. It’s not just my conclusion it’s somebody else who I’ve confided in who has also concluded these things. As they say two heads are better than one. So that gets me through because then I don’t think I’m alone going through the motions, there is somebody else that understands what I’m going through and is there to support and give advice.”* P02038 *“I wouldn’t mind reducing again, but if I need to and [psychiatrist] agreed to it or more like suggested it really, because I think I need to keep a strong dose up really to make any difference… I’d leave it up to them to reduce it or increase it really…they’re the professionals and they know what they’re doing.”* P01040 P6005: *The study has actually helped a bit because I know a lot more about reducing my medication now than I did before. But in the future I would just reduce with my doctor and I would just try and use what I’ve learnt in the study to help guide me and I would hope that my doctor would be supportive of my wanting to reduce my medication. My current doctor that I have is supportive of that so there is hopefully going to be the option to continue to reduce.* I: *So you feel confident about approaching your doctor if you wanted to reduce?* P6005: *Yes.* I: *And do you think, have you always felt confident to ask your doctor or do you feel more or less confident after being in the Radar study?* P6005: *I would say more confident after being in the Radar study.* *“I want to do it myself because none of the persons [were] looking at it that I have to reduce it. I didn’t tell them too much because they don’t know and they never ask me these questions and stuff …. Why are you not letting me choose what I want to do with me?... The study definitely came with the idea that I can stop it and I can actually have will to do it and have my own rights to do it.”* P02014

### Effects of antipsychotic reduction/discontinuation

Participants typically described effects as more significant in the latter stages of medication reduction.

#### Reduction of negative effects

Most participants (n = 21) described reduced adverse effects with dose reductions. Reductions in sedative effects and increased energy levels were most common, alongside cognitive changes including improvements in concentration, alertness, mental clarity, fluency of speech, and reductions in emotional blunting. Some described more specific impacts including reductions in increased appetite and hypersalivation, and less commonly improvements in sleep, agitation and anxiety.

*“I get tired in the morning and then just to get up is a struggle when I was taking full dose. But when I reduced it, I was able to get up in time and then I was full of energy.”* (P01022)

Indirect impacts of reduced negative effects were also described. For example, reduced appetite and sedation led to weight loss, increased ability and motivation for daily activities, better mobility, or improvements of chronic health conditions (e.g., “*It’s a lot easier to breathe being lighter*”, P03004). Some described how greater mental clarity and motivation reduced anxiety levels as they felt better able to regulate their responses to everyday challenges, with positive knock-on effects for social functioning.

#### Returning aspects of self and social functioning

Some participants described a broader return of psychological and social aspects of the self that they felt had been suppressed by antipsychotics. This manifested in various ways including improved self-confidence, more authentic connections with others, better social integration and functioning, and a return of creativity or sense of humour. Some experienced a substantial shift in self-identity linked to a forgotten enthusiasm for life, and a sense of optimism for a more active and normal future.

*“I feel more like myself I think. Even when it’s negative I still feel like I can recognise where it’s coming from, it’s part of me… Sometimes when I feel angry… it’s a natural thing that comes up from realising something in my life that I’ve been lacking…. It’s the material to improve actually, I feel like more aspects of me are open”* (P14002)

#### Challenges with emotional intensity

Although reductions in overall numbing or mental clouding brought benefits for many, often the intensity, rawness or ‘realness’ of emotions as antipsychotic levels reduced were challenging or experienced as a mixed blessing. Reducing antipsychotics was described by some as losing an emotional buffer to cushion life stressors or difficult events. Low mood, increased anxiety, and strong feelings of anger or regret were described. For some, being able to think more clearly, combined with the return of emotions led to rumination and feelings of grief and loss relating to a history of mental health problems and their impacts.

*“It’s tolerable when you are still taking it but then when you stop taking it you feel too much and I think that’s why I was drinking to try and cover that feeling up … But I think the fact that it protects your nerves from feeling creepy or anxious or something like that then all those feelings come back when you come off the medication … I think it made me feel more free and young but at the same time this feeling that your nerves are exposed and vulnerable to attack. So it was more of a feeling of excitement came back because that excitement isn’t there as much when you are on the medication and you don’t know how to deal with it”* (P06004)

#### Mental health deteriorations and relapse

Over half of participants described negative impacts of antipsychotic reduction on their mental health, consisting of the return of specific psychotic symptoms, increases in emotional intensity or other symptoms. This was sometimes preceded by a period in which the person was primarily aware of positive impacts of early dose reductions. Nine people experienced a psychotic relapse (as defined by the trial endpoint committee). Five were defined as severe relapses (requiring hospitalisation), four were non-severe (managed in the community). Many described negative impacts of mental health deteriorations on social functioning or family relationships. Participants typically described a combination of sleep problems, agitation or anxiety, and life stressors preceding relapses. For some, increased emotional intensity morphed into paranoia and psychotic symptoms. Often antipsychotic doses were increased, or reductions paused at this point.

*“When it went down to 20 I started having a problem with sleepless nights. I was having a hard time because I wasn’t sleeping for four nights a week. I was very down in health and struggling and frantic. I think I did talk to [psychiatrist] about it because I told him I felt people were trying to harm me and they’d put something in my food which was keeping me awake.”* (P5006)

#### Short-term difficulties/withdrawal effects

Some participants described short-term physical or mental effects following dose reductions that resolved after a few weeks, indicative of withdrawal effects. These including shakiness, sweating, skin crawling, dizziness, poor sleep, low mood, anxiety, emotional intensity and psychotic-like symptoms, and were reported most by those who made the largest reductions or discontinued entirely. Some compared these to experiences of coming off recreational drugs, some framed these as withdrawal effects, and some made linkages with dose reductions that they thought had been too rapid.

*“I persevered and it worked…yes, a bit shaky at first [after reducing] but that’s all gone away... I did get shivers, shakiness, and things like that but they went away after two weeks… I was getting a bit down, depressed, maybe because I’m used to having all the tablets in my system for years… but on 75mg I’m alright now, I’m better mentally, everything is fine.”* (P02026)

### Making sense of reduction experiences: the RADAR trial as a novel potential learning context

Participants made sense of antipsychotic reduction/discontinuation experiences over the 24-month period by weighing up benefits and challenges across the broad domains described above. This was shaped by each person’s profile of medication reduction (see Fig. 1), and embedded within social circumstances, personal priorities, relationships with services and existing beliefs about health, mental health and medication. We identified variations in forms of learning within the trial as an underlying theme in participants’ sense-making. Some experienced medication reduction within clinical care as a unique opportunity that facilitated new perspectives. Around half the sample described reduction experiences as personally valuable in some way, despite substantial challenges including return of psychotic symptoms or relapses. Many valued fewer adverse effects, a more active or ‘normal’ life or a more complete sense of self. Reduction-related difficulties were often framed as part of a learning journey that increased agency, understanding and confidence. A sense of optimism, ownership or empowerment often characterised these narratives:

*“Even though I get really low days, even though I get overwhelmed, even though I can’t sleep I am excited about life again.[…]It sounds a bit heavy, but I don’t feel like I want to die anymore…it’s just normal emotions… I wake up in the morning and… I’m not feeling tired, I’m not feeling like a zombie.[…]That’s so different for me. I haven’t been doing that for the last 10 years. I wasn’t interested in anything before.[...]I want to live and even though it’s difficult I can’t give up now.”* (P06015)

In contrast, for around a third of participants, the trial had not felt substantially different from regular clinical care and there was less evidence of changed perspectives or additional forms of personal engagement. These people described fewer positive impacts of reduction, and less general impact on their lives. Some appeared not to have reached an overall position on their experiences of antipsychotic reduction, and some appeared pessimistic or lacking hope.

*“I probably have felt pretty much the same in between [reductions in medication] so a bit disappointed it wasn’t a miracle cure.[…]I didn’t feel like a new lease of life or anything… with anything that’s to do with my mental health struggles and medication I take to try and help, I want it to make it better, make things stop and not just continue to suffer really… Sometimes I manage to function and sometimes I don’t really…they’ve [antipsychotics] not made it go away or cured me that’s definite.”* (P01037)

We identified three specific domains in which trial experiences shaped forms of understanding and learning, described below.

#### Medication: learning about the implications of reduction

For many participants experiencing lower medication doses helped them understand the role, impacts, and value of medication for them personally or ascertain a personally optimum dose. Several thought that without the trial their psychiatrist would not have supported this. Some extended these insights into differentiating between medication effects, their illness, and their underlying self-concept. A sense of having more options about medication was significant or empowering for some, and many were highly engaged with reduction processes and detailed reflections about dose levels.

*“From 20 down to 15 or a lower dose wasn’t so difficult… anything below 10 was more difficult for me... I had a lot of anxiety when I went down to 5 and 2.5… I wasn’t feeling so good on those doses… It’s hard to explain how I felt, just uncomfortable in myself, lack of self-satisfaction, more nervous, more wary, more alert of the outside world. I’d say things become more real like my problems and my aspirations. Everything becomes a lot more real, a lot more serious… 7.5 is OK but sometimes I don’t feel as good on 7.5 as I would say with more of a dose.”* (P12005)

Other participants—some of whom valued antipsychotics for symptom management or framed their mental health problems in biomedical terms–found reductions difficult to tolerate as psychotic symptoms returned or increased. Not continuing further with medication reductions (as permitted within the trial protocol) to avoid further mental health deteriorations meant that, for some, trial participation did not appear to impact substantially on their personal relationship with medication.

Experiential learning shaped views about future medication use with expectations of discontinuation generally diminished among those who relapsed. Some pivoted from strong opposition to antipsychotics to greater acceptance or ownership linked to awareness of their vulnerability to stressors and the potential for relapse.

*“The relapse was not very helpful, that may not have happened without the [discontinuation of] medication so that may be the only negative about it. Having said that, without that happening… I would not be on the lower dose now…this dose seems to be a dose that helps me to concentrate, and my words are not as difficult to choose under this dose under 10mg. I really came to the conclusion that this is something I can live with.”* (P07006)

Many for whom trial reductions had been personally significant were optimistic for more flexible medication use. Some were hoping to reduce further or stop their medication in the future, or felt trial experiences had initiated a longer-term personal learning journey about medication. Others for whom the trial had been less impactful thought there may not be significant longer-term implications.

#### Self-management

New perspectives on self-management and the interaction of medication with other support for mental health were also apparent. Here again, there was diversity. Some described their symptoms as unpredictable, appeared to struggle to understand their condition, or conveyed a sense that they were not able to shape or control their mental health. Other interviews featured detailed personal understandings of mental health problems and their management (which sometimes pre-dated trial participation). Many participants viewed their mental health as requiring on-going support, with medication complementing self-management strategies. Some described how trial experiences (both reductions and conversations with clinicians) had increased their awareness of triggers or early warnings of deteriorating mental health.

*“I’m not so against taking medicine but I also feel like I’ve learnt a lot over the past two years on how to make sure I don’t relapse rather than depending on the medicine, it’s actually how I choose to think and what decisions I make and different stuff.”* (P02001)

A range of self-management techniques to overcome the challenges of medication reduction and support well-being were reported including distraction, practical or emotional support from friends or family and strategies to understand or manage voice hearing and other symptoms. Others reported few self-management strategies and sources of support outside formal mental healthcare. A few people used alcohol to dampen uncomfortably intense emotions, but this had often precipitated worsening mental health or relapse. Life events, stressors or social isolation had disrupted stability or made medication reductions harder for several. A few described additional support, guidance and monitoring from clinicians, but for the most part sources of support and self-management strategies were independent of mental health service providers.

#### Relationships with clinicians: partnership possibilities

Some participants described interactions with psychiatrists as differing from those they had experienced previously when they had not been offered options to reduce medication. For many, open discussions with prescribers about medication reduction and its impacts had enabled new dynamics that felt more partnership-like, equal or trusting and were experienced as confidence-building or empowering. Medication reduction was described by some as a joint experiment with a clinician whose guidance and monitoring provided reassurance.

*“Having confided my thoughts and feelings with the psychiatrist we reached the same sort of conclusion and that was like the reduction was good... So, at that time I thought to myself… I’m doing well fighting off the voices and ignoring them. If I’m able to maintain that I might be able to reduce further.”* (P02038)

Others described few changes and a more traditional care model in which they deferred to clinical expertise. In a few cases contact with prescribers and services was reduced during the pandemic or became strained due to loss of trust, such that reduction-related learning occurred primarily independent of clinical support. Two participants disengaged from clinical services during the trial, both of whom discontinued medication, suffered severe relapses and were subsequently subject to coercive treatment.

Views about future relationships with clinicians were similarly diverse. Some felt more confident to ask about future mediation changes than previously. Most said they would not go against medical advice, although a few expressed wanting to stop medication even if their psychiatrist did not support this. Others were only interested in future reductions if initiated by their psychiatrist.

## Discussion

Our study highlights diverse and changing profiles of self-described impacts of antipsychotic reduction/discontinuation over 24 months across physiological, psychological and social domains. For some participants, and at some points during the trial, positive impacts of reduction were experienced including increased energy, improved mental clarity or weight loss, sometimes leading to greater self-confidence, levels of activity or social connection, or more positive sense of self. For others or at other points, difficulties were experienced, particularly emotional intensity, broader mental health deteriorations, return of psychotic symptoms or relapse. The experience of losing an emotional buffer was particularly significant, with struggles with low mood, anxiety and anger described. Difficulties or withdrawal effects following dose reductions were commonly reported. Each person’s trajectory was unique resulting in interview narratives characterised by optimism, pessimism, agency, defiance, passivity or combinations of these. For some, medication reduction within the trial constituted a personally significant opportunity to learn about their responses to different medication doses and develop self-management strategies. Despite sometimes substantial mental health challenges including relapses, some participants felt generally empowered or more confident in how they understood their mental health problems and medication, and related to prescribers. Others described less overall impact or benefit from trial participation. Some had initial hopes for medication discontinuation but revised these as they struggled with progressively lower doses. Others were less initially motivated by medication reduction at the start of the trial which may have translated into lower levels of personal engagement with reduction processes or stronger preferences to pause or stop medication reductions if symptoms reappeared.

Reported reductions of negative effects of antipsychotics are consistent with previous work on experiences of antipsychotics.^2^^,^^3^ Our findings also highlight struggles with a range of intense emotions as a common difficulty of dose reduction. This has been reported less but is consistent with suggestions that antipsychotics often dampen emotions.^19^ The trial context of our work meant that those who may not otherwise have chosen to reduce medication were included, and allowed exploration of experiences when reductions were endorsed and guided by clinicians using gradual and flexible reduction protocols.

While some participants showed similarly highly levels of engagement and self-determination to studies of people who chose to reduce medication, often independent of clinicians,^8^^,^^9^^,^^12^ others relied more passively on guidance from clinicians, mirroring previous work on interactions about medication between people with recurrent psychosis and clinicians.^20^ Reducing medication openly with a clinician was a novel experience for many participants, welcomed by some but not all. Some changed dynamics with prescribers related to increased confidence or openness about medication were described. These ranged from a greater sense of cooperation or partnership with prescribers, to (for a minority) increased tensions or rifts with prescribers and services relating to different opinions about medication.

A number of research and clinical implications are suggested by this work. The diversity in how people made sense of medication reduction experiences in interview narratives likely reflects both different trial experiences (physiological, psychological and clinical impacts of dose reductions) and personal preferences, motivations and values. Teasing out the factors at play is difficult and further research is needed to explore sub-groups in our sample and whether these relate to previously identified groups whose acceptance of medication and relationships with prescribers vary.^20^, ^21^, ^22^ Why some participants experienced learning and empowerment but others appeared not to should be investigated further.

Findings have implications for clinicians considering how best to manage antipsychotics, particularly given the prevalence and poor outcomes of discontinuing medication abruptly without clinical support.^7^ Some participants exemplified a position compatible with shared decision-making (SDM) that is recommended across healthcare.^23^ In SDM psychiatric consultations are conceptualised as discussions between two differently positioned experts, with service users contributing treatment preferences and experiential expertise.^24^^,^^25^ Experiencing the impacts of reducing or stopping medication helped many participants enhance this expertise and develop views on their optimal medication dose, in line with good practice guidelines.^26^^,^^27^ Others preferred to defer to clinicians’ decisions or had poor relationships with prescribers or services, illustrating some of the challenges of realising SDM in mental health.^28^

Findings complement those of the main trial RADAR trial^16^ in helping service users and clinicians alike develop realistic expectations of antipsychotic reduction processes and experiences. Medication reduction/discontinuation carries risks of mental health deteriorations or relapses and may not be suitable for some. Service users who wish to try dose reductions may benefit from reduced adverse effects but encounter emotional or other challenges with their mental health. Services should work with users to develop, encourage or enhance person-centred self-management techniques during and following dose reductions. The immediate and medium to long-term impacts of successive dose reductions should be carefully monitored, ideally within open partnerships with clinicians. Beyond this, our qualitative data suggests that people’s experiences of these processes and how they make sense of them is extremely variable. Despite gradual reduction protocols in the RADAR trial, withdrawal effects were reported. Awareness of these will help clinicians tailor slow and gradual dose reductions that may be better tolerated.^29^ Psychological support to help manage intense emotions experienced by many should be further developed and evaluated.

The strengths and limitations of this work should be considered. The size of the RADAR trial allowed us to construct a diverse purposive sample using several socio-demographic, clinical and trial intervention variables that may have shaped experiences. Our sample was relatively large (for qualitative studies) and included a sub-group who relapsed (n = 9, 35% compared to 41% in the trial reduction group). Interview questions encouraged reflection on the 24-month trial period and long-term impacts of medication changes (although reports may have been impacted by recall). The study team included a lived experience researcher, the RADAR principal investigator, trial manager and researchers, and a qualitative methodologist. While diverse perspectives fed into the design, conduct and analysis of interviews, and reflexive practices were adopted, the views of this team as generally supportive of medication reduction possibilities should be acknowledged. We may not have adequately captured the perspectives of those with the most negative trial experiences, or conflictual relationships with services as these people may have been less likely to agree to participate in interviews for this study.

In conclusion, this is the first qualitative study to explore experiences of antipsychotic reduction/discontinuation overseen by clinicians in a trial context. Participants reported benefits associated with reduced adverse effects of antipsychotics; challenges related to intensity of emotions, mental health deteriorations or relapses; and for some, enhanced understandings of their mental health and medication. Together with quantitative trial results,^16^ our findings add to the evidence base that clinicians and service users seeking to optimise antipsychotic medication can draw on. This suggests that gradual, clinically guided reductions of antipsychotics carry risks of mental health deteriorations or relapses but for some, may also bring psychosocial benefits and opportunities for learning.

## Contributors

The study was designed and overseen by NM and JM. JM is the chief investigator of the RADAR project of which this study is a part of. NM is a co-applicant and SP is the co-chief investigator. Recruitment was conducted and overseen by JS and data were collected by ML, SJ, RC, and JA-R. Analysis was conducted by ML, NM, and JM who all had direct access to the full data set. SJ provided a lived experience perspective on data collection and analysis. MH contributed a clinician perspective on data analysis. All other authors contributed to discussions about the analysis and presentation of findings. NM and ML wrote the original manuscript, and all authors approved the final draft and had responsibility for the decision to submit for publication. JM is guarantor of the study.

## Data sharing statement

The data for this study are restricted as they contain sensitive and potentially identifying information. For researchers who meet the criteria for access to confidential data, requests for access should be made to Prof. Joanna Moncrieff, University College London, at j.moncrieff@ucl.ac.uk.

## Declaration of interests

NM has grants from NIHR, Economic and Social Research Council, and North East London NHS Foundation Trust. RC is an unpaid Board Member of the International Institute for Psychiatric Drug Withdrawal (IIPDW), has undertaken paid work for the All Party Parliamentary Group for Prescribed Drug Dependence, and is a paid advisory board member for the PARTANE Study. MH is co-applicant on grants from the Medical Research Future Fund (MRFF) in Australia. He has received consulting fees from Outro Health, a digital clinic aimed to support people to stop unnecessary antidepressants; and lecture fees from NHS Trusts for grand rounds presentations, Salomon’s University and University of Washington. He sits on the DSMB of the RELEASE trial in Australia and is a co-founder of Outro Health. He is a member of the Critical Psychiatry Network and an Associate of the International Institute of Psychiatric Drug Withdrawal (both unpaid roles). JM has grants from NIHR and is a co-applicant on grants from the Medical Research Future Fund (MRFF) in Australia. She receives royalties from six books about psychiatric drugs, and has received lecture fees from Alberta Psychiatric Association, British Psychological Association, Universite de Sherbrooke, Case Western Reserve University and University of Basal. She is co-chairperson of the Critical Psychiatry Network and a board member of the Council for Evidence-based Psychiatry (both unpaid roles). All other authors (ML, SJ, JA-R, JS and SP) declare no competing interests.
