# Supplementary material for: Experiences of reduction and discontinuation of antipsychotics: a qualitative investigation within the RADAR trial
Source: eClinicalMedicine. 2023 Sep 28;64:102135. doi: 10.1016/j.eclinm.2023.102135 (PMC10626156; doi:10.1016/j.eclinm.2023.102135)
Supplement: Appendix 1 [file mmc1.docx]

Appendix 1: COREQ 32-item Checklist

Authors: 1. Nicola Morant; 2. Maria Long; 3. Sandra Jayacodi; 4. Ruth Cooper; 5. Johura Akther-Robertson; 6. Jacki Stansfeld; 7. Mark Horowitz; 8. Stefan Priebe; 9. Joanna Moncrieff.

| **Number** | **Item** | **Description** |
| --- | --- | --- |
| **Domain 1: Research team and reflexivity** | | |
| 1. | Interviewer | Authors 2, 3, 4, and 5 were members of the core team of researchers on the ‘Research into Antipsychotic Discontinuation and Reduction’ (RADAR) randomized controlled trial and conducted interviews. Author 3 is a lived experience researcher. Interviewers were supported and supervised by authors 1, 6 and 9. |
| 2. | Researcher credentials | Author 1: PhD  Author 2: BA (Hons.), PGCert, MSc  Author 3: LLB (Hons.)  Author 4: BSc (Hons.), MSc, PhD  Author 5: BSc  Author 6:BSc (Hons.), MSc, PhD  Author 7: BA, BSc, GDPsych, MSc, MBBS (Hons.), PhD  Author 8: Dipl.-Psych., Dr. med. habil., FRCPsych  Author 9: MBBS (Hons.), MD |
| 3. | Occupation | Author 1 is an associate professor specializing in qualitative research in mental health. Author 2 is a research fellow specializing in qualitative and mixed methods mental health services research. Author 3 is a lived experience peer researcher and has experience of reducing and discontinuing antipsychotics. Author 4 is a research associate specializing in mixed methods research, who has conducted research into strategies to minimize antipsychotic use. Author 5 is a senior research coordinator and researcher with experience in mixed methods research. Author 6 was the programme manager for the RADAR trial and an experienced mixed methods researcher. Author 7 is a clinical research fellow and expert in reducing psychiatric drugs. Author 8 is a professor, psychologist and medical doctor, qualified in psychiatry, who is the co-principal investigator of the RADAR study. Author 9 is a professor, consultant psychiatrist, expert on psychiatric medication and the principal investigator of the RADAR study. |
| 4. | Gender | Authors 1, 2, 3, 4, 5, 6 and 9 are female. Authors 7 and 8 are male. |
| 5. | Experience and training | All authors are active researchers with experience and training in qualitative, quantitative, and mixed methods approaches. Authors have researched and published in the broad topic area previously. Author 1 is an applied qualitative methodologist working in the field of mental health. |
| 6. | Relationship established | Participants were drawn from the pool of people recruited from secondary care mental health services and allocated to the reduction arm of the RADAR randomized controlled trial. Authors 2, 4, and 5 collected quantitative data for the trial and had contact in this capacity with some of the participants who were interviewed for the current study. Selected participants were invited to take part by Authors 2, 4 and 5, and the study was briefly presented. Interested participants were sent information, had the opportunity to consider participation and were invited to ask questions before completing written informed consent. Interviews were conducted in pairs with at least one lived experience researcher (Author 3 and another lived experience interviewer), in order to facilitate rapport building and enrich data collection. |
| 7. | Participants’ knowledge of the interviewer | As discussed above, Authors 2, 4 and 5 had contact with some of the participants in the current study in their capacity as researchers on the RADAR trial. Participants were informed about who the involved researchers were in the Participant Information Statement (PIS). They were informed in the PIS and before the interview that we were interested in hearing all their views about being in the RADAR trial including more negative experiences. |
| 8. | Interviewer characteristics | In most cases, interviews were conducted in pairs with Authors 2, 4 and 5 paired with Author 3 and another lived experience researcher. Training in qualitative methods was provided by Author 1, and ongoing support during data collection was provided by Authors 1, 6 and 9. Characteristics of interviewers are covered in items 1-3 above. We report on our reflexive approach in relation to the positioning of the author team in the ‘data analysis’ section of the Methods and the ’strengths and limitations’ section of the Discussion. |
| **Domain 2: Study design** | | |
| 9. | Methodological orientation and theory | The study adopted an overall epistemological position of critical realism. This guided the design and conduct of semi-structured interviews and our analytic strategies and evaluations of these. We used codebook thematic analysis, an approach described as ‘medium Q’ (Braun and Clarke, 2021, p242). Analysis combined inductive and deductive approaches, allowing us to both explore our initial research questions and capture the experience and concerns of our research participants. Codebook thematic analysis was used to generate a combination of ‘topic summary’ and ‘shared meaning’ themes. |
| 10. | Sampling | We used purposive sampling with a view to capturing diversity in intervention delivery, pre-trial medication factors and participant characteristics. Data from the main RADAR trial were used for this: demographic and clinical data were collected at baseline and 24 months; data on antipsychotic medication use and other clinical factors including relapse were collected at two monthly intervals during the trial. We aimed to obtain a diverse sample that included variations in the following: ethnic background, age, gender, employment status, relapse status during the trial, antipsychotic medication reduction profile, whether the reduction intervention was delivered by a RADAR affiliated doctor or a local clinician, NHS Trust (trial site), years in contact with mental health services, antipsychotic polypharmacy and administration (depot/oral) and clozapine use. In considering minimum numbers for each of these, we compared with their broad prevalence in the main RADAR trial sample.  To obtain our interview sample, members of the central RADAR team (Authors 2, 4, 5 and 6) identified participants as they completed their final 24-month follow-up who fulfilled the sampling framework criteria. Later stages of sample construction involved reviewing the sampling characteristics of already interviewed participants and identifying characteristics that had been captured less so far. We then targeted invitations to participate to people who met these criteria to ensure that we obtained a broadly balanced sample. |
| 11. | Method of approach | Following the initial process of identifying potential interview participants described in 10, local researchers then contacted these trial participants to briefly present the current study. If the person was in agreement, a member of the interviewing team (Author 2, 4 or 5) then contacted potential participants to discuss the study in more detail and respond to any questions. For those who were interested in taking part an information sheet, consent form, visual representation (timeline) of their antipsychotic medication use in the trial, and basic topic prompts to aid recall of the process of reduction/discontinuation were sent in the post. Written informed consent was received before the interview was conducted. |
| 12. | Sample size | 26 |
| 13. | Non-participation | Thirty-four participants were invited to take part. Of these, eight did not participate for the following reasons: two did not have capacity to take part (possible dementia diagnosis, relapse in mental state); two did not want to take part in research; one had a recent bereavement and one preferred not to take part in any further RADAR research due to negative experiences of the trial; and two chose not take part after reading the participant information sheet. This resulted in 26 people who participated in interviews. No-one withdrew their data after taking part. One interview was not completed in full due to participant communication difficulties. |
| 14. | Setting of data collection | Initially data collection was conducted in participants’ preferred settings as far as possible: in participants’ homes and in NHS community mental health buildings. After the national restrictions were introduced as part of the covid-19 pandemic, interviews were conducted over the phone and secure video conferencing software. |
| 15. | Presence of non-participants | Participants could bring others along to interviews but did not choose to do so. |
| 16. | Description of sample | Twenty-six participants drawn from the reduction/discontinuation arm of the RADAR trial (n=126 in total), with recurrent non-affective psychosis recruited from secondary care mental health services across eleven Trusts in England.  . |
| 17. | Interview guide | Interview questions included: effects and impacts of the antipsychotic reduction/discontinuation programme, experiences of relapse, sources of support, experiences of intervention delivery. The interview topic guide is included in Appendix 2. |
| 18. | Repeat interviews | n/a |
| 19. | Audio/visual recording | Interviews were audio-recorded and transcribed verbatim. |
| 20. | Field notes | Authors 2, 3, 4 and 5 who conducted the interviews wrote short reflexive summaries soon after completing each interview. These were designed to capture their impressions of the emotional and positional feel of each interview encounter. Reflexive summaries were anonymized and shared with other members of the team. They were used to inform decisions about when data collection should stop and in analysis to complement transcripts and provide a contextual overview of each interview to aid understanding of specific segments of text. |
| 21. | Duration | Individual interviews lasted 30-90 minutes. |
| 22. | Data saturation | Regular meetings were held within the co-author team to discuss data collection. Interviews were continued until the team considered that sufficient richness and diversity of data had been obtained. This was discussed and agreed upon by all authors. |
| 23. | Transcripts returned | Transcripts were not returned to participants. |
| **Domain 3: Analysis and ﬁndings** | | |
| 24. | Number of data coders | Author 2 led the analysis, with close input from author 1. Author 3 provided a lived experience perspective, contributed to early coding, and participated in later analytic discussions. All other authors were involved in analytic discussions with some contributing at early stages, and others at later stages relating to data presentation in the final write up. |
| 25. | Description of the coding tree | Descriptions of the themes, subthemes and codes were developed and captured in an iteratively developed coding framework in Nvivo software. The presentation of Results and structure of two main sections and sub-sections within these is based on this. However, due to the analytic focus and word limit of the paper, this does not reflect our full coding framework. |
| 26. | Derivation of themes | Our analysis took a hybrid inductive and deductive approach. Following the broad principles and stages of thematic analysis, data codes, themes, and subthemes were iteratively developed and refined throughout the analytic process. Analysis involved collaborations between co-authors, with meetings held during both data collection and data analysis phases. Co-authors discussed their impressions of the data, initial and developing thematic ideas, and provided critical feedback and commentary on early drafts of analytic writing. |
| 27. | Software | NVivo 12 |
| 28. | Participant checking | While participant checking did not take place, Author 3 (lived experience researcher) wrote reflexive notes following interviews, was involved in analytic discussions, and coded a proportion of transcripts alongside Author 2. |
| 29. | Quotations presented | Indicative quotes are included within the manuscript and in table 2. All identifiable information has been removed and quotes are attributed to interviewees via a unique code. |
| 30. | Data and findings consistent | Data presented in indicative quotes in the Results section and table 2 are chosen to illustrate how the themes, experiences and concepts described in the analytic text were manifested in interview narratives. A statement about data sharing is included at the end of the paper. |
| 31. | Clarity of major themes | Results are presented in two broad sections that cover our two main thematic domains. The first section, (“Effects of antipsychotic medication reduction / discontinuation”), presents findings on the reported effects of antipsychotic reductions / discontinuations. This material is primarily topic based, designed to show the reader the range of reduction experiences reported by participants when encouraged to express this in their own words. In the second section of the Results (“Making sense of reduction experiences: the RADAR trial as a novel potential learning context”), we present more meaning-based analytic findings. Here, as signalled in the section title and described in the first paragraph of this section (p7), the central organising concept is “forms of learning”.  The decision to include a topic summary theme was driven by awareness that the impacts and experiences of antipsychotic reduction processes reported by participants feature important and novel descriptions that required little interpretation. |
| 32. | Clarity of minor themes | Each of the two main sections of the Results are divided into sub-sections. In the first section, (“Effects of antipsychotic reduction / discontinuation”) sub-sections relate to specific topic sub-domains that comprise the important direct experiences and indirect impacts or changes described by participants. Sub-section titles are designed to capture the nature of the changes experienced:   - Reduction of negative effects - Returning aspects of self and social functioning - Challenges with emotional intensity - Mental health deteriorations and relapse - Short term difficulties/ withdrawal effects   In the second main section (“Making sense of reduction experiences: the RADAR trial as a novel potential learning context”) each of the sub-sections presents how diversity in forms of learning is manifested in relation to medication (section 2.1), self-management strategies (section 2.2), and relationships with clinicians (section 2.3). These sub-sections focus on different areas of experience, but their common focus is on what was learned (or not) through participants’ sense-making of reduction experiences in the trial. Again, sub-section titles are designed to capture the nature of these intra and interpersonal learning experiences:   - Medication: learning about the implications of reduction - Self-management - Relationships with clinicians: partnership possibilities   Within each sub-section, analytic text describes both commonalities and variations across participants, and notes the nature and extent of divergences. |
