# Supplementary material for: Experiences of reduction and discontinuation of antipsychotics: a qualitative investigation within the RADAR trial
Source: eClinicalMedicine. 2023 Sep 28;64:102135. doi: 10.1016/j.eclinm.2023.102135 (PMC10626156; doi:10.1016/j.eclinm.2023.102135)
Supplement: Appendix 2 [file mmc2.pdf]

Antipsychotic reduction programme participant interview indicative topic guide

Preparation

Data will be identified in advance of the interview from the RADAR study database about participants' antipsychotic reduction profile. This data will be used to shape the questions in this interview.

Introduction and orientation to topic

The interview will be understood to be a discussion between the researcher and participant. Begin with an introduction about why you are here, reminder about the programme. Let people know what you are going to do with the data. Reassure people that you are not coming to assess them or their psychiatrist or anyone from their mental health team. You want to know if there are any ways that we can make the programme better.
"I am here today to talk to you about your experiences of the RADAR antipsychotic reduction programme, about whether the programme was helpful to you, or not. The research team will use the information you give us today to think about how we can develop this programme, so it is very helpful for us that you have agreed to talk to us."
If difficulties with recall, provide information about the programme so that participant is orientated to the topic of conversation. It would be useful to have a copy of the individual reduction schedule as part of the programme with you to help guide the conversation and orientate people to what is being discussed.
As we talk today, there are no right or wrong answers at all – we are interested in your views and experiences."
Take tape recorder out:
"*I would like to record our conversation today - is that OK? That's so I can I really listen to what you say."* [If refuses tape recorder, ask for permission to take notes]
"*Once I leave here today, our conversation will be typed up. At no time will your name or personal details be attached to the written version of our conversation or to any materials we publish."*

Overview / Opening: ***"Can you tell me about your antipsychotic medication use over the last two years?"***

Optional probes to use as necessary:

- How long have you been taking antipsychotic medication?
- What did you think of your [X medication] at the start of the study?
- How has your [X medication] been more recently (eg.in the last 12 months)?

If participant reduced:

2. What it was like to [reduce X medication]. Can you tell me a bit about that please."

- Tell me about the first few months when you began to [reduce X medication]. What was that like?
- How much did you manage to reduce?

- Did you stop completely? [If they have reduced/stopped] – how did you manage to reduce/stop, what was that like?

If participant did not reduce/ reduced only a little:

- Did you not reduce at all or only a little? If so why do you think that was?
- Aim to probe both positives and negatives about medication and reduction. If need to encourage person to elaborate, use ‘why’ / ‘tell me a bit more about that’ questions.
- General picture of their mental health over the 2 year RADAR period. Any periods when you ‘felt well / became unwell’ / when things didn’t go so well?

3. Questions on impacts (if not already covered in Qn 1 response): ***“Can you tell us how it made you feel to be taking less of X?”***

- Optional probes to use as necessary (for each area try to get a sense of both positive and negative effects if possible):
- General effects
- Symptom effects
- Emotional / mood effects
- Physical health effects
  Impacts on daily functioning, i.e. everyday tasks
  Impacts on relationships (“getting along with”): partner, family, friends, wider social network. Whether people close to them noticed any differences / had / have any views on the impacts of their medication changes.

4. Exploration of relapse / negative experiences / periods of being unwell that seem attributable to antipsychotic reduction: ***“Have there been any periods of time over the last 2 years whilst you’ve been in the RADAR Study when you’ve been more unwell than usual? Can you tell me a bit more about that please?”***

Optional probes to use depending on the person’s response to the main question and information already explored earlier in the interview:

- What happened?
- Were there any particular difficulties taking place in your life during that time, eg relationships, work, benefits, housing etc?
- Any additional involvement of mental health services?
- Get a sense of where the person was in reduction / withdrawal process at the time
- What happened during / after this period re: antipsychotic reduction.
- How long did this episode last
- Were there any broader impacts in the person’s life
- How did they feel about it
- If the person has described one episode in detail, probe whether there were any other negative experiences / periods of being more unwell over the 24 month period.
- Why do you think you became unwell?

5. Contact with and support from mental health services during RADAR. ***"I'd like to ask you now about how it was seeing [your psychiatrist / Dr X] who managed your [X medication] during the RADAR study."***

- What was it like [reducing X medication] with Dr X?
- Did it feel different in any way to your meetings with Dr X before RADAR?
- Did you see Dr X more often or for longer than you'd previously seen him/her/a psychiatrist?
- Did you feel able to raise any concerns / problems you had about the medication / reducing medication [and how did Dr X deal with these?]
- Other mental health practitioners seen during RADAR. Whether this was different from / more than previous contacts and how they found this.
- Probe about *relationships* with both the prescribing psychiatrist, and other mental health practitioners, and whether these felt different from 'normal' / before in any ways.
- Anything they would like to have been different about this contact / support from mental health services.

6. Other sources of support for antipsychotic reduction: ***"Can you tell me about anything else you found helped you when you were [reducing X medication]. This might be people, or organisations, or things that you do yourself".*** NB: As much as possible, try to keep this discussion focussed on support specific to medication reduction, as opposed to support in general.

Optional areas to probe as necessary:

- Other health service or third / voluntary sector support / contact, e.g. talking therapy
- Support / participation in local community
- Internet-based support
- Well-being / health / self-management / exercise
- Spiritual / religious
- Family / friends

7. Recommendations and Changes: ***"Overall, looking back at your experiences of [reducing X medication], what would you say to someone else thinking about [reducing their antipsychotics]?"***

Areas to probe:

- Overall, how would you balance the good things and the difficulties you've experienced?
- Would you recommend [reducing antipsychotic medication] to other people and why?
- What did you find the most difficult thing about [reducing X medication]?
- What might have made it easier for you (particularly for people who report difficulties / not having reduced much / becoming unwell)
- What changes would you like to see to how [reducing X medication] is done.

8. How was taking part in the RADAR study?

Optional probes to use as necessary:

- Can you remember why you decided to take part in the RADAR study
- What did you hope to get out of it?
- How did you find being part of a research study, was there anything you liked or didn't like?
- How did you find seeing researchers and doing assessments? How did you feel about the length of the assessments? Did you find that any of the questions we asked you were difficult?
- How did you feel about the payment?

9. Feelings and attitudes towards antipsychotic medication in the future*. "Now that you have taken part in the RADAR study, how do you feel about taking antipsychotics in the future?"*

Areas to probe:

- (If didn't reduce at all/much) How would you feel about reducing your antipsychotic medication in the future? If so, is there anything that would help you with this?
  - *Refer to probes in question 6 - support, services etc*

- (If reduced down to a low dose but did not discontinue) How would you feel about further reductions to your medication in the future? If you would like to do this, is there anything that would help you with this?
  - *Refer to probes in question 6 - support, services etc*

- (Regardless of circumstance) How would you feel about approaching your psychiatrist to discuss antipsychotic medication changes in the future? Would you feel differently about this after taking part in the RADAR study?

Close: ***"Do you have anything to add about any of the topics we've discussed today? Anything else you'd like to say that you think is relevant that we haven't talked about."***

Thank people for their time and contribution to the programme. Re-state confidentiality and anonymity of data.
